# Boosting psychological well-being in infertile women undergoing IVF through Fordyce happiness counseling: a quasi-experimental approach in Zahedan, Southeast Iran

**DOI:** 10.1016/j.pmedr.2025.103372

**Published:** 2026-01-04

**Authors:** Tahereh Boryri, Parisa Delghavi, Somayyeh Khazaeian

**Affiliations:** Pregnancy Health Research Center, Midwifery Department, Zahedan University of Medical Sciences, Mashahir Square, Zahedan 9816743463, Sistan and Baluchestan Province, Iran

**Keywords:** Infertility, In Vitro Fertilization (IVF), Psychological distress, Fordyce's approach

## Abstract

**Objectives:**

Infertility imposes a significant psychological burden, especially for women undergoing assisted reproductive technologies(ART). This study assessed the effectiveness of Fordyce's happiness-based counseling in reducing depression, anxiety, and stress among women undergoing in vitro fertilization (IVF).

**Methods:**

A quasi-experimental study was conducted among 60 women (30 intervention, 30 control) undergoing IVF in Zahedan, Iran, in 2024. Infertility clinics affiliated with Zahedan University of Medical Sciences were randomly selected, and eligible participants were recruited based on inclusion criteria. The intervention group received six weekly face-to-face counseling sessions based on Fordyce's happiness model. Psychological outcomes were assessed using the Depression Anxiety Stress Scale-21 (DASS-21) before and four weeks post-intervention. Data were analyzed using independent *t*-tests, chi-square tests, and repeated measures ANOVA.

**Results:**

Counseling significantly reduced stress (F = 181.4, *p* < 0.01, partial η^2^ = 0.8), anxiety (F = 29.3, *p* < 0.01, partial η^2^ = 0.3), and depression (F = 125.1, p < 0.01, partial η^2^ = 0.8). Post-intervention mean scores in the intervention group decreased for stress (18.5 ± 2.9), anxiety (17.6 ± 2.3), and depression (21.5 ± 5.7) compared with control scores (26.6 ± 5.1; 24.8 ± 1.9; 24.8 ± 5.1).

**Conclusion:**

Fordyce's happiness-based counseling is an effective, low-cost, culturally adaptable intervention to alleviate psychological distress in women undergoing ART. Integrating it into infertility care programs may enhance emotional well-being, particularly in resource-limited settings.

## Highlights

• The program significantly reduced depression, anxiety, and stress in women undergoing in vitro fertilization.
• Participants in the intervention group reported improved psychological well-being.
• Fordyce's happiness counseling proved effective, low-cost, and culturally adaptable.
• Findings support integrating happiness-based counseling into infertility care services

## 1 Introduction

Infertility is clinically defined as the failure to achieve pregnancy after 12 months of regular, unprotected sexual intercourse (Zegers-Hochschild et al., 2017). It affects approximately 8–12 % of reproductive-aged couples worldwide (Shen et al., 2024). It has increasingly been recognized not only as a biomedical issue but also as a significant psychological and social challenge, particularly among women (Deyhoul et al., 2017). Infertility is commonly categorized as either primary—where conception has never occurred—or secondary—where couples fail to conceive despite previous pregnancies (Vander Borght and Wyns, 2018). In Iran, a recent meta-analysis reported primary and secondary infertility rates of 18.3 % and 2.5 %, respectively, both exceeding global averages (Deyhoul et al., 2017). Despite notable advances in assisted reproductive technologies (ART), women undergoing infertility treatments often experience substantial emotional distress, particularly symptoms of anxiety and depression (Abdollahpour et al., 2022). Studies have reported that between 52 % and 76 % of infertile couples suffer from psychological disorders (Hamzehgardeshi et al., 2019; Kiani et al., 2021), which can detrimentally impact their quality of life and reduce the effectiveness of fertility treatments (Keetharuth et al., 2018). These findings underscore the importance of incorporating psychological support into ART protocols (Sax and Lawson, 2022). Among the various non-pharmacological approaches, the Fordyce Happiness Program has emerged as a promising cognitive-behavioral intervention designed to enhance psychological well-being (Mohammadi et al., 2024). This structured program comprises 14 principles—8 cognitive and 6 behavioral—designed to promote healthy personality traits, reduce unnecessary worries, lower unrealistic expectations, cultivate authenticity, and ultimately increase subjective happiness (Fordyce, 1977; Forutannasab et al., 2024). By reshaping cognitive and emotional responses to life challenges, this approach facilitates more adaptive coping strategies(Rabiei et al., 2020). Although psychological interventions have shown efficacy in reducing emotional distress in women undergoing in vitro fertilization (IVF) (Hamzehgardeshi et al., 2019; Rahimi et al., 2021). The application of the Fordyce Happiness Program in infertility care in Iran remains limited. This gap is particularly relevant in culturally unique and underserved areas such as Sistan and Baluchestan Province. In this region, the prevalence of secondary infertility reaches 12.9 %—significantly higher than the national average—while primary infertility is reported at 2.7 % (Nasrabad et al., 2024). The compounded effects of psychological distress, cultural stigma, and restricted access to mental health services highlight the urgent need for context-sensitive, scalable mental health interventions.

This study is designed based on the following question: Does counseling based on Fordyce's happiness approach significantly reduce depression, anxiety, and stress levels in infertile women undergoing IVF treatment compared to standard care?

## 2 Methods

### 2.1 Study design and population

This quasi-experimental study employed a pretest–posttest design and was conducted between June and December 2024 in Zahedan, Iran. The study population consisted of infertile women attending Infertility Clinics affiliated with Zahedan University of Medical Sciences (ZUMS). Zahedan is the capital of Sistan and Baluchestan Province, located in southeastern Iran. ZUMS operates two infertility clinics in different areas of the city. Using randomization software (www.random.org), one clinic was randomly assigned to the intervention group and the other to the control group.

**Fig. 1:**
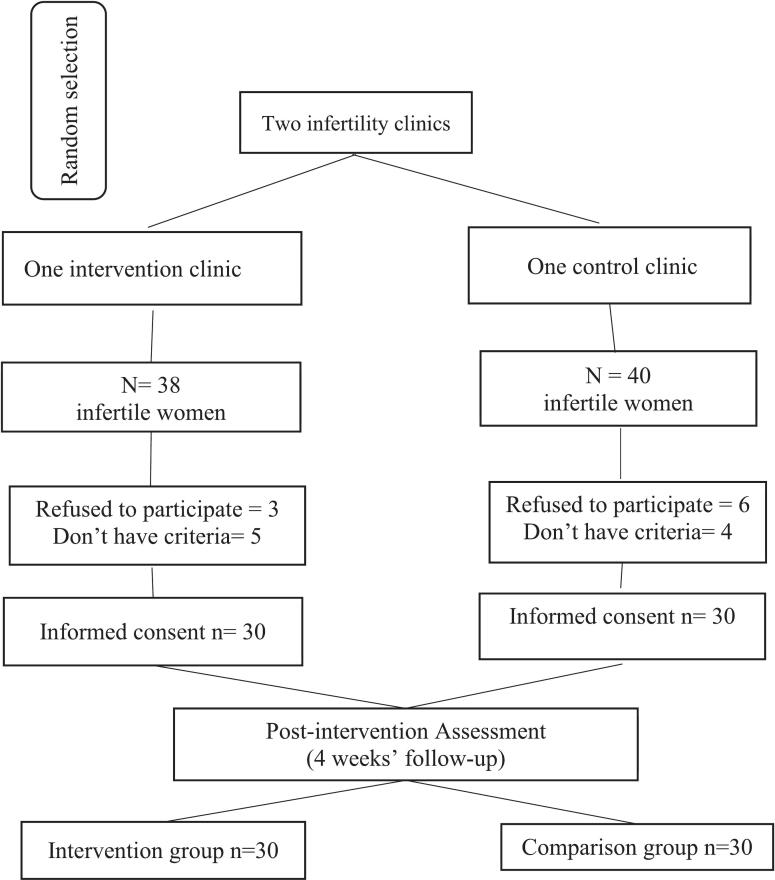
Flow chart of study participants, including the random selection of infertility clinics, and selecting participants into intervention and control groups., Zahedan, Iran, June – December 2024.

Eligible participants were identified through clinic medical records and were randomly contacted and screened based on predefined inclusion criteria. Women who met the eligibility criteria and provided written informed consent were enrolled. If a selected individual declined participation or did not meet eligibility criteria, another participant was recruited from the list. After enrollment, participants were instructed to attend their respective clinics to complete baseline questionnaires (Fig. 1).

Inclusion criteria were: age 18–45 years, primary infertility with at least one year of infertility history, first-time candidates for assisted reproductive treatment, minimum literacy (ability to read and write), no substance addiction, no diagnosed psychological disorders or use of psychotropic medications, no underlying medical conditions requiring medication, and no prior participation in psychological counseling programs. Exclusion criteria included withdrawal of consent, exposure to severe stressful events within the previous six months, cancellation of infertility treatment by a physician, pregnancy occurrence during the study, absence from any intervention sessions, participation in yoga classes for six months or longer, and incomplete questionnaire responses exceeding 10 %.

Sample size was calculated using Cochran's formula (Cochran, 1977) based on anxiety scores reported in a previous study (Abadi et al., 2019). Considering a 20 % potential attrition rate, a total sample size of 60 participants (30 per group) was determined.

z1−α2+z1−β2s12+s22x¯1−x¯22

Study parameter: alpha = 0.05, power = 0.90, ${\bar{x}}_{1}$=4.4, ${\bar{x}}_{2}$=7.6, S_1_ = 2.0, S_2_ = 4.6, estimated sample sizes' = 60 so N per group = 30.

The intervention group received six weekly face-to-face counseling sessions based on Fordyce's happiness model. Each session lasted 45–60 min and was conducted in small groups of seven to eight participants at the infertility clinic. Participants received summary booklets after each session and were contacted twice weekly to address questions or concerns. The control group received routine care during the study period and was provided with educational materials after completion of data collection.

This study was approved by the Ethics Committee of Zahedan University of Medical Sciences (IR.ZAUMS.REC.1403.113). Written informed consent was obtained from all participants, confidentiality was ensured, and participants were free to withdraw at any time. The study was conducted in accordance with the Declaration of Helsinki.

### 2.2 Measures

The questionnaire consisted of questions related to contextual, midwifery variables. In this section, the variables were designed quantitatively or qualitatively.

#### 2.2.1 Depression Anxiety Stress Scale-21 (DASS-21) questionnaire

The Depression, Anxiety, and Stress Scale-21 (DASS-21) was employed to assess the emotional states of participants. This self-report instrument comprises 21 items, each rated on a four-point Likert scale ranging from zero (never) to three (almost always). DASS-21 is divided into three subscales of seven items each, measuring depression, anxiety, and stress. The depression subscale includes items three, five, 10, 13, 16**,** 17, and 21. Anxiety is evaluated through items two, four, seven, nine, 10, 15, and 19, while stress is assessed using items one, six, eight, 11, 12, 14, and 18. Scores for each subscale are calculated by summing the relevant item scores and multiplying by two to align with the original 42-item DASS-21. Interpretation of scores follows these guidelines: Depression: zero–nine (normal), 10–13 (mild), 14–20 (moderate), 21–27 (severe), ≥28 (extremely severe). Anxiety: zero–seven (normal), eight–nine (mild), 10–14 (moderate), 15–19 (severe), ≥20 (extremely severe). Stress: zero–14 (normal), 15–18 (mild), 19–25 (moderate), 26–33 (severe), ≥34 (extremely severe) (Hoseini-Esfidarjani et al., 2022).

This study utilized the Persian version of the DASS-21, which has been previously validated for use in Iranian populations (Hoseini-Esfidarjani et al., 2022; Najafi Kalyani et al., 2013; Sahebi et al., 2005). In the present study, the Cronbach's alpha for the entire DASS-21 questionnaire was 0.9, indicating high internal consistency. The depression, anxiety, and stress subscales all demonstrated satisfactory reliability, each with a Cronbach's alpha of 0.8.

### 2.3 Statistical analysis

Descriptive statistics, including frequencies, percentages, and mean ± standard deviation, were used to summarize the research data. To compare baseline variables between the two groups, *t*-tests and chi-squared tests were employed. The effectiveness of the intervention was evaluated using repeated measures analysis of variance (ANOVA), which assessed differences between pretest and posttest scores within each group. Effect sizes were calculated using partial eta squared (η2), where values of 0.01, 0.06, and 0.14 corresponded to small, medium, and large effects, respectively (Tabachnick et al., 2013). Data normality was assessed using the Shapiro–Wilk test. All statistical analyses were performed using SPSS version 24.0 (IBM Corp., Armonk, NY, USA). All statistical tests were two-tailed, and a *p*-value <0.05 was considered statistically significant.

## 3 Results

This study involved 60 eligible infertile women, divided into intervention and control groups. The demographic and contextual characteristics of the participants are detailed in Table 1. According to the findings, the mean age of participants in the intervention group was 28.4 ± 6.6 years, while in the control group, it was 27.8 ± 6.2 years. The duration of infertility was reported as 5.3 ± 0.5 years for the intervention group and 4.6 ± 0.6 years for the control group. The most prevalent cause of infertility in both groups was female factors. Statistical analysis revealed no significant differences between the two groups regarding demographic and contextual characteristics (*P* < 0.05) (Table 1). Table 2 presents the mean scores of stress, anxiety, and depression before and after follow-up in both intervention and control groups. According to the findings in Table 2, a majority of participants in both groups reported severe stress levels before the intervention: 60 % in the intervention group and 53.4 % in the control group. Regarding anxiety levels, most participants exhibited extremely severe symptoms: 83.4 % in the intervention group versus 90 % in the control group. For depression, extremely severe levels were observed among 53.3 % of participants in the intervention group and 40 % in the control group before initiating interventions (Table 2). The independent *t*-test revealed no statistically significant differences between the two groups in terms of the mean scores of stress (*P* = 0.14), anxiety (*P* = 0.25), and depression (*P* = 0.31) before the intervention. The repeated measures ANOVA was utilized to examine changes over time and between and within groups (Table 3, Table 4). The results showed a significant interaction effect between group and time for stress (F = 116.4, *P* < 0.01, partial ƞ2 = 0.7), anxiety (F = 14.6, P < 0.01, partial ƞ2 = 0.2), and depression (F = 23.4, P < 0.01, partial ƞ2 = 0.1) (Table 3). Furthermore, within the intervention group, significant improvements were observed over time for stress (F = 140.3, P < 0.01, Partial ƞ2 = 0.8), anxiety (F = 437.5, P < 0.01, Partial ƞ2 = 0.6), and depression (F = 526.8, P < 0.01, Partial ƞ2 = 0.8). Conversely, no significant changes were detected in the control group (Table 4).

**Table 1:** Baseline characteristics of infertile women, Zahedan, Iran, June – December 2024.

Variable	Intervention group (n = 30)	Comparison group (*n* = 30)	*P* value	Total (*n* = 60)
	Mean ± SD/n (%)			
Age	28.4 ± 6.6	27.8 ± 6.2	0.72^a^	28.1 ± 6.1
Marriage Length	6.1 ± 0.6	5.1 ± 0.4	0.36^a^	5.6 ± 0.5
infertile Length	5.3 ± 0.5	4.6 ± 0.6	0.28^a^	4.9 ± 0.5
Women's Educational Status				
Primary, secondary	12(40)	10(33.3)	0.47^b^	22(36.6)
Middle & upper	18(60)	20(66.4)		38(63.4)
Spouse's Educational Status				
Primary, secondary	10 (33.3)	8(26.7)	0.23^b^	18(30)
Middle & upper	20 (66.4)	22(73.3)		42(70)
Women's Employment Status				
Housewife	23(76.7)	22(73.3)	0.76^b^	45(75)
Employee	7(23.3)	8(26.7)		15(25)
Spouse's Employment Status				
Unemployed	3(10)	4(14.3)	0.50^c^	7(11.6)
Employee	27(90)	26(85.7)		53(88.4)
Causes of infertility				
Male factors	8(26.7)	6(20)	0.53^b^	14(23.3)
Female factors	13(43.3)	14(46.7)		27(45)
Combined or unknown causes	9(30)	10(33.3)		19(31.7)
Satisfaction with income				
Sufficient	5(16.7)	6(20)	0.56^b^	11(18.3)
No sufficient	12(40)	13(43.3)		25(41.6)
Fairly sufficient	13(43.3)	11(36.7)		24(40.1)
Covered by Insurance				
Yes	25 (83.3)	27 (90)	0.35^c^	52(86.6)
No	5 (16.7)	3 (10)		8 (13.4)

*Footnotes:* standard deviation (SD); frequencies (percentages), n (%); a independent *t-*test; b chi-square test; c Fisher's exact test.

**Table 2:** Descriptive Statistics Before and After Intervention in intervention and control groups, Zahedan, Iran, June – December 2024.

Variables	Intervention group(n = 30)		Control group (n = 30)	
	pretest	Post-test	pretest	Post-test
	Mean ± SD/ n (%)			
**Stress**	28.4 ± 5.8	18.5 ± 2.9	26.3 ± 4.9	26.6 ± 5.1
Mild (15–18)	0	13(43.3)	0	0
Moderate (19–25)	12(40)	17(56.7)	14(46.6)	12(40)
severe (26–33)	18(60)	0	16(53.4)	18(60)
Extremely Severe (≥34)	0	0	0	0
**Anxiety**	23.6 ± 5.4	17.6 ± 2.3	25.6 ± 3.9	24.8 ± 1.9
Moderate (10–14)	0	5(16.6)	0	0
severe (15–19)	5(16.6)	15(50)	3(10)	2(6.6)
Extremely Severe (≥20)	25(83.4)	10(33.4)	27(90)	28(93.4)
**Depression**	26.4 ± 5.8	21.5 ± 5.7	24.9 ± 5.9	24.8 ± 5.1
Moderate (14–20)	7(23.3)	14(46.7)	8(26.7)	7(23.3)
Severe (21–27)	7(23.3)	16(53.3)	10(33.3)	13(43.3)
Extremely Severe (≥28)	16(53.3)	0	12(40)	10(33.4)

Mean scores ± standard deviation (SD) and frequency distributions (n (%)) for stress, anxiety, and depression before and after intervention in control and intervention groups. **Notes:** pretest = before intervention, post-test = after intervention.

**Table 3:** Pre- and post-test score differences by intervention and control groups over time, Zahedan, Iran, June – December 2024.

Variable	Intervention group (n = 30)		Control group (n = 30)		Time effect			Group × time interaction effect		
	pretest	Post-test	pretest	Post-test	F	P	Partial ƞ^2^	F	P	Partial ƞ^2^
	Mean ± SD		Mean ± SD							
Stress	28.4 ± 5.4	18.5 ± 2.9	26.3 ± 4.9	26.6 ± 5.1	181.4	<0.01	0.8	116.4	<0.01	0.7
Anxiety	23.6 ± 5.4	17.6 ± 2.3	25.6 ± 3.8	24.8 ± 1.9	29.3	<0.01	0.3	14.6	<0.01	0.2
Depression	26.4 ± 5.8	21.5 ± 5.7	24.8 ± 5.9	24.8 ± 5.1	125.1	<0.01	0.8	23.4	<0.01	0.1

*Footnotes:* pretest = before intervention; post-test = after intervention. Partial eta squared (η^2^) was used as a measure of effect size. F denotes the F-statistic of the analysis of variance. All probability values (P) were obtained from repeated measures analysis of variance.

**Table 4:** Within-group differences in pre- and post-test scores, Zahedan, Iran, June – December 2024.

Variable	Intervention group (n = 30)			Control group (n = 30)		
	F	P	Partial ƞ^2^	F	P	Partial ƞ^2^
Stress	140.3	<0.01	0.8	0.4	0.54	0.0
Anxiety	437.5	<0.01	0.6	2.1	0.15	0.0
Depression	526.8	<0.01	0.8	245.7	0.06	0.0

*Footnotes:* Partial eta squared (η^2^) was used as a measure of effect size. F denotes the F-statistic of the analysis of variance. All probability values (P) were obtained from repeated measures analysis of variance.

## 4 Discussion

This study demonstrated that Fordyce's happiness-based counseling produced substantial reductions in depression, anxiety, and stress among women undergoing IVF compared with standard care. These findings align with previous research documenting the effectiveness of happiness-focused and positive psychology interventions in mitigating psychological distress and enhancing mental well-being among infertile women. Psychological distress itself is known to predict IVF outcomes, highlighting the clinical importance of addressing emotional burden in this population (Aimagambetova et al., 2020). Recent evidence using the DASS-21 similarly reports elevated levels of stress and anxiety among women experiencing reproductive challenges, including recurrent pregnancy loss (Issakhanova et al., 2023). A growing body of research supports the beneficial role of psychosocial and cognitive-behavioral interventions in improving the psychological state of infertile women. Positive thinking and cognitive-reconstruction counseling have been shown to significantly reduce perceived stress among women with previous failed IVF cycles (Masoumi et al., 2025). Likewise, Mindful Self-Compassion therapy has demonstrated meaningful reductions in depression, anxiety, and stress (Sahraian et al., 2024), while other interventional studies have reported improvements in psychological well-being and marital satisfaction among infertile couples (Sepahvand et al., 2023). More broadly, stress-management programs incorporating relaxation, guided imagery, and supportive counseling have also yielded significant improvements in stress and self-esteem during IVF treatment (Damghanian et al., 2025; Gupta et al., 2024; Sepahvand et al., 2023; Shafaghi et al., 2024). Consistent with these findings, happiness-enhancing interventions have been shown to reduce symptoms of depression, anxiety, and stress in women with a history of recurrent miscarriage (Elsharkawy et al., 2021). Despite such promising results, evidence on the long-term sustainability of psychosocial interventions remains mixed. A recent systematic review and meta-analysis concluded that while psychological interventions may strongly enhance well-being outcomes, their effects on reducing distress are more modest and less consistent (Jackson et al., 2025). Similarly, another recent meta-analysis focusing on cognitive behavioral therapy (CBT) for infertile women reported significant reductions in depression and anxiety and improvements in quality of life after intervention (Yıldız Karaahmet et al., 2025). Such variability in long-term outcomes may reflect differences in sample size, intervention content, cultural or contextual factors, baseline distress levels, or follow-up duration. Nevertheless, positive psychology–based approaches remain appealing due to their low cost, accessibility, and minimal risk (Wen et al., 2020). Fordyce's Happiness Program integrates cognitive restructuring, present-moment awareness, and structured practices such as gratitude exercises to cultivate positive emotions, strengthen psychological resilience, and reduce unrealistic expectations (Forutannasab et al., 2024). By promoting positive emotional states and enhancing coping skills, these strategies help individuals regulate emotional responses and reduce stressors contributing to anxiety (Rabiei et al., 2020). Regular engagement in happiness-enhancing activities has also been associated with sustained improvements in depression and overall well-being (Yıldız Karaahmet et al., 2025). In the present study, the Fordyce program led not only to reductions in mean DASS-21 scores but also to clinically meaningful improvements in symptom severity. Most participants in the intervention group shifted from severe to milder categories of distress, whereas those in the control group experienced minimal change. These findings underscore the practical value of happiness-based interventions for alleviating emotional distress among women undergoing IVF and highlight their potential role in comprehensive infertility care.

This study has several strengths, including its focus on a psychologically vulnerable population and its implementation in a region with a high prevalence of infertility, which enhances the contextual relevance of the findings. Nevertheless, certain limitations should be acknowledged. The quasi-experimental design, non-random sampling, limited recruitment to public university clinics, and short follow-up period constitute the main limitations of this study. These factors could introduce selection bias and affect generalizability, although key baseline variables such as age, education, and infertility history were standardized to mitigate this effect. Future randomized controlled trials with longer follow-up and broader sampling are warranted to confirm and expand upon these results.

## 5 Conclusion

The findings of this study demonstrate the significant effectiveness of Fordyce's Happiness Program in reducing depression, anxiety, and stress among infertile women undergoing IVF treatment. By addressing the unique emotional challenges of this vulnerable population, the intervention enhances psychological resilience and overall well-being, leading to meaningful improvements in mental health and quality of life. These results highlight the importance of integrating structured, culturally sensitive psychological support into infertility care, particularly in the high-stress context of IVF. Such counseling could be delivered through group sessions or individual sessions led by a nurse or midwife, integrated into existing IVF programs. Healthcare providers and policymakers should prioritize the development and implementation of sustainable, accessible interventions tailored to the cultural characteristics and specific needs of this population.

## CRediT authorship contribution statement

**Tahereh Boryri:** Writing – review & editing, Methodology, Formal analysis, Conceptualization. **Parisa Delghavi:** Writing – review & editing, Supervision, Methodology, Formal analysis, Data curation, Conceptualization. **Somayyeh Khazaeian:** Writing – original draft, Resources, Project administration, Methodology, Investigation, Formal analysis, Data curation, Conceptualization.

## Funding

No specific funding was obtained for this study.

## Declaration of competing interest

The authors declare that they have no known competing financial interests or personal relationships that could have appeared to influence the work reported in this paper.

## Data Availability

The datasets generated and/or analyzed during the current study may be available from the corresponding author on reasonable request.
